# Causal interactions in resting-state networks predict perceived loneliness

**DOI:** 10.1371/journal.pone.0177443

**Published:** 2017-05-18

**Authors:** Yin Tian, Li Yang, Sifan Chen, Daqing Guo, Zechao Ding, Kin Yip Tam, Dezhong Yao

**Affiliations:** 1 Biomedical Engineering Department, ChongQing University of Posts and Telecommunications, ChongQing, China; 2 Chongqing Key Laboratory of Photoelectronic Information Sensing and Transmiiting Technology, Chongqing, China; 3 Chongqing High School Innovation Team of Architecture and Core Technologies of Smart Medical System, Chongqing, China; 4 Key Laboratory for NeuroInformation of Ministry of Education, School of Life Science and Technology, University of Electronic Science and Technology of China, Chengdu, China; 5 Faculty of Health Sciences, University of Macau, Taipa, Macau, China; Institute of Psychology, Chinese Academy of Sciences, CHINA

## Abstract

Loneliness is broadly described as a negative emotional response resulting from the differences between the actual and desired social relations of an individual, which is related to the neural responses in connection with social and emotional stimuli. Prior research has discovered that some neural regions play a role in loneliness. However, little is known about the differences among individuals in loneliness and the relationship of those differences to differences in neural networks. The current study aimed to investigate individual differences in perceived loneliness related to the causal interactions between resting-state networks (RSNs), including the dorsal attentional network (DAN), the ventral attentional network (VAN), the affective network (AfN) and the visual network (VN). Using conditional granger causal analysis of resting-state fMRI data, we revealed that the weaker causal flow from DAN to VAN is related to higher loneliness scores, and the decreased causal flow from AfN to VN is also related to higher loneliness scores. Our results clearly support the hypothesis that there is a connection between loneliness and neural networks. It is envisaged that neural network features could play a key role in characterizing the loneliness of an individual.

## Introduction

One of the basic needs of humans is to enjoy a sense of belonging in social groups [1]. From an evolutionary point of view, humans engaging with social groups can benefit from shared resources and security because social isolation is more likely to lead to serious challenges in survival. If a discrepancy occurs between expected social relations and actual social relations, people could feel loneliness, which is generally defined as a negative emotional response to the experience of this discrepancy [2]. Eventually, maintaining stable social (interaction) relationships (a stable social network) evolved into an adaptive goal, which influenced human’s behavior, cognition and emotion [3–5]. Lack of or insufficient social integration may result in adverse trends, including self-deception and negative emotion [3,6]. In particular, loneliness represents an unpleasant subjective mental experience in connection with qualitative or quantitative deficiencies within an individual’s social network [7]. Loneliness influences emotional and cognitive processes. Being in a lonely situation for a long time could lead someone to develop a serious personality disorder or other mental illness, which may result in suicide, cognitive impairment and an increase in the risk of an early onset of Alzheimer's disease [8–10].

Loneliness entails the absence of a sense of belonging, and lacking a sense of belonging has been argued to underlie the above-discussed negative effects [3,6]. When individuals think that their status in their social networks is decreased because of some threat, they are motivated to improve their status to an acceptable degree [11]. Lonely individuals experience an unsatisfactory social life and are likely to feel a chronic lack of belonging. Their unmet belonging needs trigger lonely individuals to continue trying to improve their social relationships [12]. Social monitoring, which is activated by increased belonging needs, can be used for this purpose [13,14]. Generally, people can prevent rejection and increase inclusion by monitoring their environment for social cues [15]. Thus, paying more attention to social cues could facilitate decoding of social cues, which further strengthens social monitoring [3,14]. Two conflicting assumptions were proposed to explain the mechanisms of loneliness: increased social monitoring and decreased social monitoring. For the former, it was presumed that lonely individuals should engage in increased social monitoring for insufficient belonging when compared to non-lonely individuals [3]. However, it is also possible that lonely individuals may exhibit deficient belonging as a result of a lower level of social monitoring when compared to non-lonely individuals. A low level of social monitoring would reduce the attention of lonely individuals to social cues, thus leading to difficulties in decoding social cues, such as poor emotion recognition skills. Regardless of which mechanism is at work, attention is thought to be in some manner a key factor in loneliness.

Some researchers have found that lonely individuals have a lower level of social cue perception, leading to a lower level of social skills and reduced communication [4,16–18]. This lower skill level may originate from a low level of attention to decoding social cues. To compensate, lonely individuals have to enhance their response to exogenous stimuli for achieving a safe feeling level while attempting to ignore the perception of social cues for the endogenous stimuli. The ability to reorient attention from an ongoing action in response to potentially dangerous and threatening triggers is critical for human survival. Here, the ventral attentional network (VAN), mostly lateralized to the right hemisphere including the inferior frontal gyrus (IFG), the temporal parietal junction (TPJ), and the anterior insula (AI), plays an important role in the interactions between endogenous (i.e. top-down, goal-directed) attention and exogenous (i.e. bottom-up, stimulus-driven) attention [19]. Reorienting attention to behaviorally related sensory stimuli is a major function of the VAN [19,20]. When the dorsal attentional network (DAN) is activated by goal-directed tasks, the VAN is suppressed so that people can focus on their ongoing targets to prevent attention shifting from unrelated stimuli. However, top-down attention will be interrupted to deal with external emergency if the VAN is activated by exogenous but target-related stimuli [19]. It has been pointed out that the TPJ is triggered not only by attentional reorienting [20–23] but also by social cognition such as loneliness [4,24]. It has also been reported that there are small differences in the TPJ’s activity in response to social pictures versus non-social pictures in lonely subjects, whereas the differences in non-lonely subjects are large, which is in line with self-protection behavior in the former [4].

Emotion recognition, a component of the ability to decode social cues, was also consistently involved in loneliness according to previous studies [12,14]. Some findings suggested that lonely individuals may devote little attention to decoding social cues, leading to further weakening of emotion recognition abilities [12]. However, the results reported from different groups did not appear to be consistent with each other [3,12,25,26]. Research using fMRI imaging technology in conjunction with social and non-social emotional image recognition [4] has reported that significant activation by non-social emotional images can be seen in the ventral striatum and dorsal medial frontal gyrus (dmPFC) in young lonely cohorts. Related fMRI studies revealed that altruism exhibited widespread activation at dmPFC [16,27]. These results suggested that the lonelier the individual feels, the more overly self-protective that individual would be, with the aim of maintaining a safe distance with others psychologically. Power’s group [16] found that during the execution of an outdoor object recognition scenarios task, strong activation is shown in the dmPFC of lonely subjects. However, significant activations in non-lonely subjects were found to show up in subcortical brain regions, which were related to happiness, reward and motivation. Activations of cortical brain regions involved in social cognition and self-representation have been reported (such as the ACC, dmPFC, superior temporal gyrus, medial frontal gyrus, precentral gyrus, TPJ, and occipito-temporal cortex) [24,28]. Socially isolated subjects exhibited negative activations in the subcortical dopamine region, which is consistent with the definition of positive reward experience with social ties in the context of psychological research. The sub-cortical regions (related to emotion) worked together with cortical regions to modulate more complex cognitive functions and enhance the top-down control of neural functions. Such relationships indicate that associated cortical regions may trigger actions in emotional regions and visual cortical areas, which may be more sensitive to process information and induce plasticity of attentional orienting [20]. These studies showed that amount of social contact has a neural basis with the corresponding behavioral characteristics to respond to specific social or non-social information. However, Power’s group [16] used implicit-priming paradigm studies without achieving consistency between the behavioral effects and neurological effects. These authors believed that this inconsistency could be due to some behavioral paradigms not being suitable to conduct in the magnetic resonance environment [16–18]. This may suggest that the use of task-related fMRI alone to investigate the neural process of loneliness could be challenging. However, resting-state (rs) fMRI studies are unlikely to be confounded by differences in the effect of performing different tasks, which could be advantageous for studying loneliness compared with the conventional task-related studies.

Results from large scale behavioral and neuroimaging studies suggest that loneliness may be related to the activation level of visual, attentional and emotion-related neural networks [20,29]. Researchers tried to use their data regarding activation levels to explain the underlying neural mechanisms of loneliness, but a consensus has not yet been achieved. In the cases where stronger activation levels have been observed in lonely individuals, that activation has been found in primary visual sensory regions. Using eye-tracking technology, it has been shown that higher loneliness was related to longer gaze towards threatening social stimuli when watching this kind of movie clips [20,30]. Moreover, higher activation was observed in the visual cortex regions using fMRI when lonely individual viewed negative social images [4]. In the cases where weaker activation levels have been found in lonely individuals, that decrement has been found in emotional- and attentional-related regions. For instance, weaker activation in the ventral striatum was observed when lonely individuals viewed positive social images, indicating that loneliness may be related to positive social cues [4]. It has been shown that a smaller volume of grey matter in the posterior superior temporal sulcus was related to higher loneliness scores with the early social perception, suggesting poor ability in loneliness may be related to the decoding of social cues [26]. These results seem to be conflicting if we simply interpret loneliness as being associated with the extent of activation of brain regions involved in social perception, e.g. stronger activation being related to enhanced social monitoring and weaker activation being related to a decreased social monitoring. Instead, loneliness was modulated by the cooperation of brain networks rather than the activation of particular brain regions. Clearly, effective connectivity (e.g. Granger causality analysis, GCA) characterizes the directional flow of information between brain regions (or networks). GCA could establish the causality relationships between activation in different regions and demonstrate patterns of regulation between regions (or networks), which should provide new insight into loneliness.

Two main factors, namely attentional allocation and emotion recognition, could affect the decoding of social cues, which can in turn influence individuals’ loneliness. Based on the above, we hypothesized that altering neural network connectivity could play a key role in shaping individuals’ loneliness. As a means of testing that hypothesis, we conducted our investigation using a holistic approach. Specifically, we utilized group ICA to extract resting-state networks including the visual network, the affective network, and attentional networks (both the dorsal attentional network and the ventral attentional network). Then directional influences among different networks were evaluated by Granger causality and correlated with loneliness scores to assess their functional relevance. Finally, we investigated whether the effective connectivity could be used to predict the lonely individual’s perceived loneliness. The results from the present study can form the basis for future investigations of neural cognitive processes in lonely individuals.

## Methods

### Participants

Participants were recruited from among students at the University of Electronic Science and Technology of China. They were first administered the UCLA loneliness scales [31]. On the basis of loneliness scores, participants were divided into high-loneliness (total score>45) and low-loneliness (total score <28) groups. To reduce the effects from other factors, the social psychological indices such as depression and anxiety independency that could contribute to loneliness were also tested. If the Self-rating depressions scale (SDS) scores were higher than 60 (i.e. moderately-severely degree on depression), the participants were excluded. Thus, the behavioral ratings included six questionnaires: the UCLA loneliness scale[31], the State-Trait Anxiety Inventory (STAI)[32], the Self-rating Depression Scale (SDS)[33], the Interpersonal Reactivity Index (IRI-C)[34], the Trust Scale[35] and the Social Support Rating Scale (SSRS)[36]. Finally, thirty right-handed participants (30 males, mean ± SD = 21.3±2.4 years, including 15 high-lonely males and 15 low-lonely males) took part in the fMRI. None of the selected participants had a history of mental or neurological problems, or had previous exposure to the fMRI. Informed consent was signed prior to the study. Participants received some monetary compensation after the experiment. All experiments were approved by the ethical committee of the University of Electronic Science and Technology of China (UESTC).

### Data acquisition and analysis

#### Data acquisition

Imaging data were collected using a 3.0T GE scanner in the UESTC MR Research Center. Subjects were required to close their eyes, lie in the scanner, keep quiet and try not to move their heads. Resting-state functional images were acquired using a single shot, gradient-recalled echo-planar imaging (EPI) sequence (TR = 2000 ms, TE = 30ms and flip angle = 90^°^). Sixteen transverse slices (FOV: 24cm, in-plane matrix: 64 × 64, slice thickness: 4mm without gap), aligned along the anterior commissure–posterior commissure (AC–PC) line, were acquired. For each subject, a total of 255 volumes were acquired. The first 5 volumes were discarded to ensure steady-state longitudinal magnetization. Subsequently, for spatial normalization and localization, a set of T1-weighted anatomic images was acquired in axial orientation using a 3D spoiled gradient recalled (SPGR) sequence (TR = 6.008ms, TE = 1.984ms, flip angle = 9°, matrix size = 256 × 256 × 156).

#### Data processing

We firstly conducted the fMRI data preprocessing. fMRI data was carried out using the Statistical Parametric Mapping software (SPM8, http://www.fil.ion.ucl.ac.uk/spm/software/spm8/). Main steps included: (1) Time-correcting: slice timing corrected differences on time among the slices during acquisition. (2) Realigning: the images were realigned to the first volume for head-motion correction (translation≤1.5mm and rotation≤1.5°). (3) Normalizing: images of each subject were normalized to the T1 structural images of individual subjects. Subsequently, functional data were warped into the standard MNI (Montreal Neurological Institute) space by EPI templates, and then resampled voxels to 3×3×3 mm^3^. (4) Smoothing: data smoothing was accomplished by convolution with an isotropic Gaussian kernel to ensure high SNR (FWHM = 8mm).

**Fig 1:**
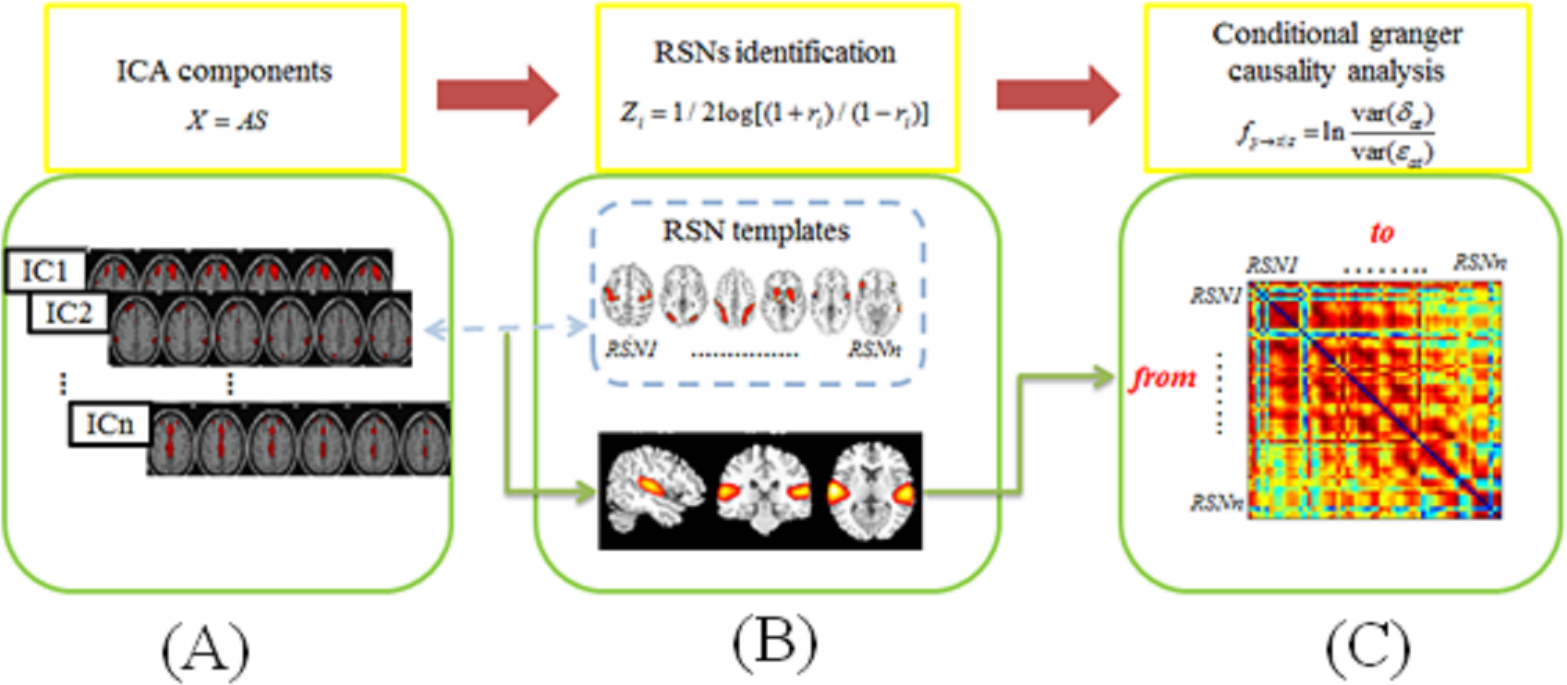
The main steps of data analysis. (A) group ICA analysis, (B) extract the four RSNs, and (C) conditional granger causal analysis

Main steps for fMRI data processing (see Fig 1) included independent component analysis (ICA), RSN identification and conditional granger causality analysis (CGCA).

The first step was ICA. The GIFT toolbox (http://mialab.mrn.org/software/gift/index.html), based on temporal concatenation from high-loneliness, low-loneliness, and all subjects and then back-reconstruction to single components, were used to conduct the group ICA, respectively. The minimum description length (MDL) criterion was used to estimate the number of independent components (ICs). Using the FastICA algorithm, an IC with time series and spatial maps was estimated after the temporal dimension concatenated fMRI data from high-loneliness, low-loneliness, and all subjects, which were then reduced to 40 using principal components analysis, respectively. The IC time series and spatial maps of each subject were back-reconstructed based on the results of group ICA. For each IC, the time series explained the dynamic processing and the spatial map indicated the intensity across the voxels for a specific pattern of brain activity. Fisher-Z transformation was used to convert the intensity of all voxels to z values with an aim to display the contribution of voxels for a particular IC. It is noted that the z-value map is a correlation between time-series of a voxel and the time-series of the entire independent components, wherein the larger the z value, the stronger the functional connectivity.

The second step was RSN identification. Based on previous investigations [26,37,38], a subset of components were selected from the 40 estimated ICs for additional analysis. Network templates [39] were obtained from functional imaging in the neuropsychiatric disorders lab (website: http://findlab.stanford.edu/functional_ROIs.html). A linear template was first used to match processing in each IC. This was accomplished by calculating the difference via the averaged z-score of voxels within the template minus the average z-score of voxels outside the template in each IC [40]. The classification of the ICs in terms of RSNs was carried out by means of the rest fMRI networks. Selected RSNs referred to the cerebral ICs with the largest spatial correlations in the network templates [41]. Based on our RSN studies, the four ICs related to RSNs can be described as follows:

*Affective network (AfN)*: Putamen, Amygdala, Olfactory, Caudate, and Pallidum [42,43].

*Visual network (VN)*: Fusiform gyrus, Calcarine fissure, Lingual gyrus, Middle occipital gyrus, Cuneus, Superior occipital gyrus, and Inferior occipital gyrus[40].

*Ventral attention network (VAN)*: SupraMarginal, inferior parietal, Angular, superior temporal gyrus, Middle temporal gyrus, inferior temporal gyrus, and Inferior frontal gyrus [44,45].

*Dorsal attention network (DAN)*: bilateral frontal eye field (FEF including the superior frontal gyrus, a portion of the precentral gyrus) and bilateral intraparietal sulcus (IPS), inferior parietal gyrus, inferior frontal gyrus, inferior frontal gyrus, middle occipital gyrus, postcentral gyrus, superior parietal gyrus, and supramarginal gyrus [44,45].

The third step was CGCA, based on a directed expansion of the autoregressive model to a general multivariate case which includes all measured variables [46–48]. Considering the case of three time-course (X_t_, Y_t_, and Z_t_), the computational formula was shown in Fig 1. Applying the theory of CGCA to the resting state fMRI data, the time-course of one of the RSNs can be associated with X_t_, and another one with Y_t_. Z_t_ represents all the remaining RSN time-courses other than X_t_ and Y_t_. Accordingly, CGCA was performed to test causal influences among RSNs using the influence terms (F_X→Y|Z_ and F_Y→X|Z_). The order of the autoregressive model was set to 1 using the Schwarz criterion (SC). The coefficients of the models were calculated using a standard least squares optimization procedure.

#### Statistical analysis

We chose four RSNs (i.e. DAN, VAN, AfN, and VN) in each group to conduct one-sample t-test to check spatial distribution. The difference of effective connectivity between RSNs was evaluated using one-sample t-tests. For each RSN, a two-sample t-test was used to test the difference between two groups (all thresholds were set at p<0.05, FDR correction). The group comparisons were masked to the voxels within corresponding RSNs. The mask was formed by integrating the regions of corresponding RSNs in both groups, which were obtained from the results using one-sample t-test. We performed Pearson correlation analysis to investigate the relationship between Z values of GA for directional influences between two networks and behavioral scores. Thresholds were set at p<0.05 (FDR correction).

If the directional influences were significantly correlated with loneliness scores, Granger causality connectivity may predict variations in feelings of loneliness of lonely individuals. Here, a support vector regression (SVR) was used to develop a predictive model for a relatively small sample size [49,50]. Leave-one-out cross validation (LOOCV) was used to test the stability of the model and the performance of the predictions [51]. Significance levels were set at p<0.05. Details of the LOOCV procedure are described as follows: We assume there are n samples in the dataset. In a typical calculation, one sample was selected as a testing set, while the rest of the samples were considered as training sets to establish the SVR model. The calculation was repeated until each sample has been assigned as a test set on one occasion. From these, n SVR models can be obtained. The correlation coefficients between predicted and actual loneliness scores were employed to evaluate the performance of the SVR model [51]. There are two advantages for LOOCV: 1) n -1 samples were used to train the SVR models, which are closest to the original distribution of the sample. This could provide a more reliable assessment of the results obtained. 2) There are no random factors affecting the training process, which ensures that the model training process is repeatable.

## Results

### Behavioral scales analysis

Pearson correlation results show that loneliness scores in both high loneliness group and low loneliness group were not significantly correlated with the other five scales (For the high-lonely group: STAI, R = -0.040, p = 0.887; SDS, R = 0.361, p = 0.186; IRI-C, R = -0.453, p = 0.090; Trust, R = -0.251, p = 0.367; SSRS, R = -0.418, p = 0.121; For the low-lonely group: STAI, R = -0.153, p = 0.585; SDS, R = -0.009, p = 0.974; IRI-C, R = 0.028, p = 0.921; Trust, R = -0.477, p = 0.072; SSRS, R = -0.203, p = 0.469). Details were also shown in Figures A and B in S1 File. These results were consistent with previous studies suggesting that loneliness and depression are separable [26,52].

### Component selection and analysis

**Fig 2:**
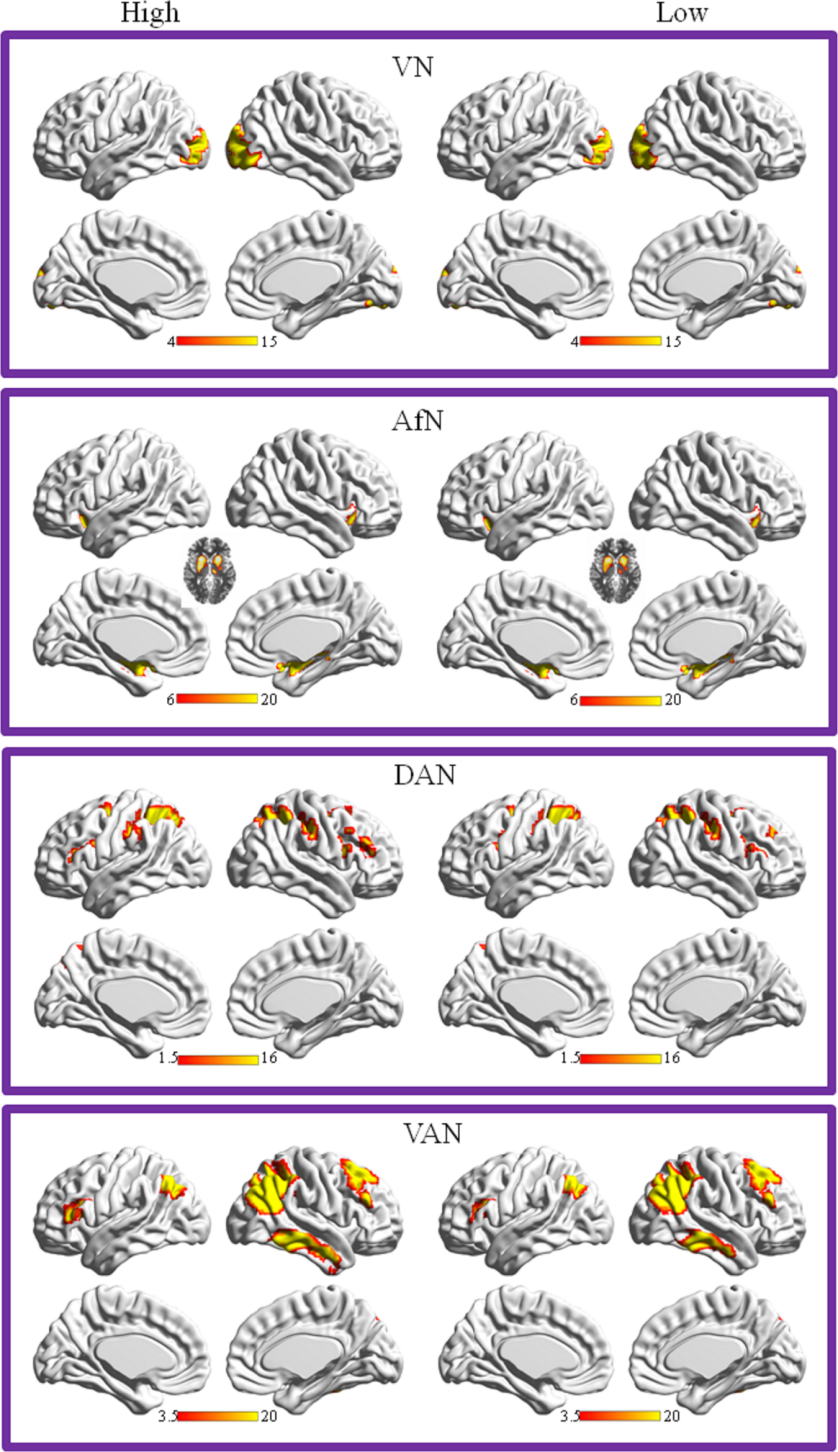
Cortical representation of the four RSNs of resting state fMRI data from a group of results across all subjects. Top view: RSNs including: affective network (AfN); Visual network (VN); Dorsal attentional network (DAN), and ventral attentional network (VAN). Bottom view: The spatial correlation coefficients of 40 ICs from all subjects with respect to the four RSN templates. The largest correlations with the templates were chosen.

The number of ICs in the resting state fMRI data was found to be 40 by using the MDL analysis. The brain network templates were obtained from the website: (http://findlab.stanford.edu/functional_ROIs.html). ICA decomposition was then applied to derive this output dimensionality. Fig 2 shows the spatial maps of the four RSNs obtained from both the high-lonely group (Fig 2 left) and the low-lonely group (Fig 2 right). The spatial correlations of the 40 ICs with respect to the four RSN templates were also shown in Figure C (high-lonely group), Figure D (low-lonely group) and Figure E (all subjects) in S1 File. In each RSN based on a one-sample t test, the active regions, the MNI coordinates of the peak foci and the associated Brodmann areas (BA) are summarized in Table A (high-loneliness group), Table B (low-loneliness group) and Table C (all subjects) in S1 File. Different brain regions between two groups were also shown in Table D in S1 File.

### Granger causality and statistical analysis

**Fig 3:**
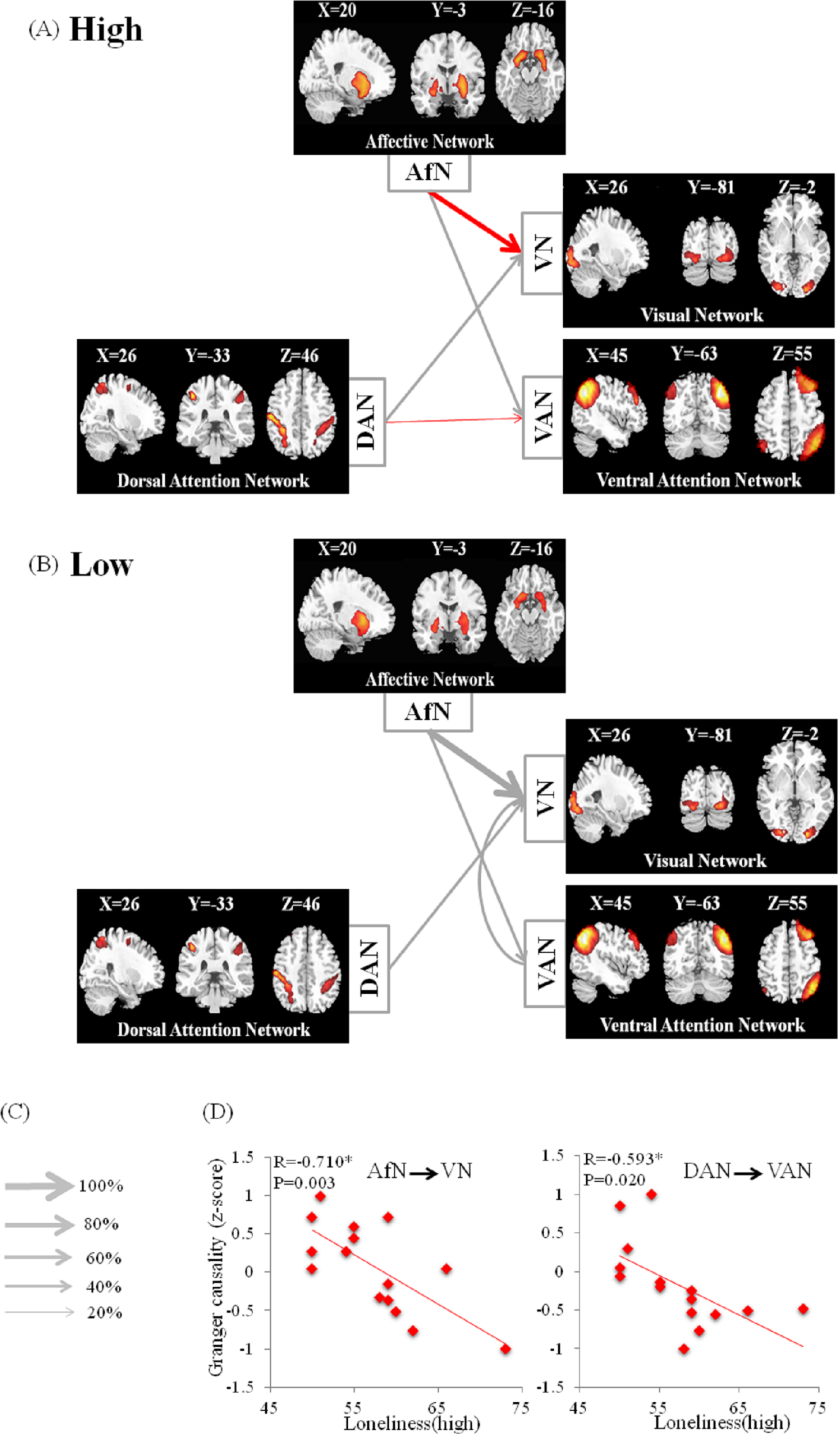
Granger causality networks between the four resting-state networks (RSNs) and correlations between loneliness scores and GC value for the CGCA. (A) lonely individuals (high loneliness scores > 45), (B) non-lonely individuals (low loneliness scores < 28), (C) The normalized scores of connectivity, and (D) granger causal influence between RSNs as function of loneliness scores. RSNs included the dorsal attentional network (DAN), the ventral attentional network (VAN), the affective network (AfN) and the visual network (VN). All negative Pearson’s correlations were observed (p<0.05, FDR correction).

Granger causal interactions between neural networks were calculated for lonely and non-lonely groups and displayed graphically with respect to the mean group z-score (see Fig 3). For both lonely and non-lonely groups, we observed significant causal flows among different networks, which are annotated as grey arrows in Fig 3 (i.e. AfN→VAN, AfN→VN and DAN→VN). Notably, the GA values and the loneliness scores showed strong negative correlations in lonely group only (see red arrows in Fig 3; AfN →VN and DAN →VAN). To examine whether the relationships between loneliness scores and GC values via CGCA analysis were specific to the loneliness scale in the lonely group, we examined GA values of AfN →VN and DAN →VAN on several other types of scales, namely, the State-Trait Anxiety Inventory (STAI), the Self-rating Depression Scale (SDS), the Interpersonal Reactivity Index (IRI-C), the Trust Scale and the Social Support Rating Scale (SSRS). The correlations between GA values and the other five scales did not reach statistical significance in the lonely group. Similarly, the six scales (Loneliness, STAI, SDS, IRI-C, Trust Scale and SSRS) did not significantly correlate with GA values in the non-lonely group. Details were also shown in Figures F-I in S1 File. Taken together, loneliness was correlated with GA values of AfN →VN and DAN →VAN only in the lonely group.

**Fig 4:**
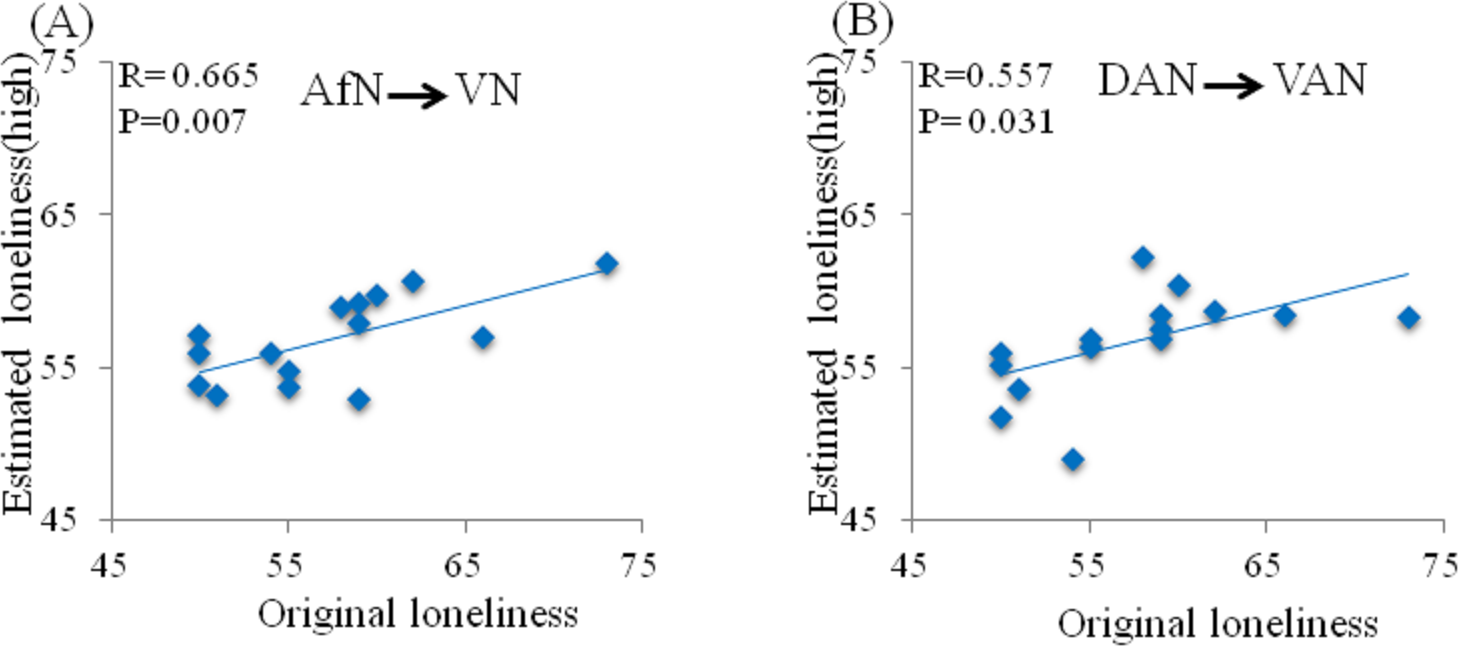
Relationships between the predicting and the original loneliness scores in lonely individual. (A) AfN→VN, and (B) DAN→VAN. The estimated loneliness scores were obtained by SVR predicting model and LOOCV.

Furthermore, SVR with linear kernel analysis and LOOCV revealed that the values of GC connectivity of AfN→VAN and DAN→VAN each significantly predicted perceived loneliness in lonely individuals. The predictive performance was evaluated by a leave-one-out cross validation scheme in conjunction with SVR. Here, the root-mean-square error of prediction (RMSEP) after checking by LOOCV were 4.885 (AfN→VN) and 5.235 (DAN→VAN), respectively. As shown in Fig 4, the predicting loneliness scores were significantly correlated with the original loneliness scores in lonely individuals (for AfN→VAN, R = 0.665, p = 0.007; for DAN→VAN, R = 0.557, p = 0.031). The results demonstrated that lonely individuals with weaker GC values of AfN→VN and DAN→VAN had higher loneliness score.

## Discussion

As far as we are aware, this work is the first investigation in which the relationship between loneliness and causality interactions between RSNs, i.e. the visual network (VN), the dorsal attentional network (DAN), the ventral attentional network (VAN), and the affective network (AfN), were evaluated using CGCA directly. We found that: (1) the causal flows from the affective network to the visual and ventral attentional networks occurred in both non-lonely and lonely individuals. Only the causal flow from the affective to the visual network was negatively correlated with loneliness scores in lonely individuals; (2) the causal flows from DAN to VN were observed in both two groups. However, only the causal flow from DAN to VAN was observed, which was generally negatively correlated with loneliness scores in the lonely group; (3) Perceived loneliness was well predicted by effective connectivity of AfN →VN and DAN →VAN.

Previous studies found that when subjects attended to a stimulus task, two attentional networks were simultaneously involved [19,53], i.e. the activated dorsal attentional network and the deactivated (suppressed) ventral attentional network, which focused on goal-directed tasks and prevented attentional reorientation from ongoing tasks (top-down information) to unrelated objects [21,23]. Clinical lesion studies where damage to VAN regions (e.g. TPJ) leads to spatial hemineglect [54] further supported the idea that the function of VAN has been assumed to reorient attention to target-related stimuli in the un-attentional focus. Researchers found that stronger causal flow from VAN to DAN was related to worse behavioral performance, indicating the action of reorienting attention [21,23,55]. It has been suggested that bottom-up signal transferred from VAN to DAN destroys the top-down attentional control, which in turn disrupts the sensorimotor processing guided by DAN [19,55]. We found that weaker DAN→VAN was significantly related to higher loneliness scores in lonely individuals. This clearly suggests a network-level mechanism whereby the decreased top-down flow from DAN made it difficult to suppress VAN activation, which prevented the filtering of behaviorally irrelevant input and enhanced the efficacy of sensorimotor processing in the attended domain [19,55–57]. Increased loneliness results in poor connectivity of the fibers within networks related to VAN, according to our previous DTI findings [20], which further substantiates the weaker causal information flow from DAN to VAN observed in the present study. Taking these together, it is likely that loneliness could be correlated to decreased suppression of VAN, which results in increased attention to interacting with the environment (i.e. by the stimulus-driven method, or reorienting). In other words, lonely individuals may favor entertaining themselves with nonsocial rewards when they feel social isolation or unsatisfied with their actual relationships [4]. As such, they are sensitive and readily adaptable to a changing environment. Therefore, lonely individuals showed low social skill, which may be due to difficulty in focusing their attention on decoding social cues[26].

Amygdala in the AfN area that linked to the orbital frontal cortex (OFC) and sensory area was believed to contribute to positive emotion and reward [58]. The amygdala guided ventral visual stream processing via attentional modulation. Moreover, evidence from VAN studies suggested that the presence of at least a third of distinctive brain circuit-cognitive processes were involved in responding to external stimuli [19] or to internal representations [59]. In consistent with these early observations, our results revealed that the causal information flows from AfN to both VAN and VN occurred in both groups, indicating that attention may be driven by task-related information, which is neither salient nor goal-directed control. Alternatively, VAN could be modulated by emotional stimuli, especially in cases where the task does not involve the processing of emotional content [60]. The current study found that the information flow from AfN to VAN occurred in both non-lonely and lonely individuals and did not relate to loneliness. It is possible that lonely individuals show increased social monitoring induced only by their increased attention to social cues, but do not represent by their skills in decoding social cues. Alternatively, focusing on social cues in socially relevant situations could be effective, because attention requires concentration and reduces executive control [61].

Previous studies found that the ventral striatum in the AfN was weakly activated by happy social pictures when compared to nonsocial pictures in lonely individuals, whereas the visual cortex in VN was strongly activated by negative social cues in lonely individuals [4]. These findings suggested that loneliness was related to brain processing of social cues (i.e. emotion recognition) [26]. However, different activation patterns were shown in different neural networks. Specifically, the decreased response to positive social pictures presented in AfN and the increased response to negative social pictures in VN were both related to loneliness. In the current study, we observed AfN→VN in both groups, but only AfN→VN was significantly negatively related to loneliness in lonely individuals, indicating that the weaker flow signal from AfN to VN was related to loneliness. Taken together, it is possible that lonely individuals showed increased social monitoring in the VN, due to the decreased information flow from the AfN. As a result, lonely individuals may devote more attention to negative social cues and ignore the positive social cues. Thus, our present results further provided the causal directional evidence in a novel perspective to explain neural mechanisms of loneliness. In particular, the different processing patterns in response to positive and negative social cues in loneliness were not due to the way in which lonely individuals attend to and decode social cues but rather to the ways in which they interpret or use those cues to gain inclusion so as to improve their feelings about their social relationships.

## Conclusion

We have explored the relationships between loneliness scores and the possible effective connectivity between RSNs, i.e. DAN, VAN, AfN and VN. We used a data-driven technology based on ICA to extract RSNs from resting-state fMRI data. Then, conditional granger causal analysis was used to obtain directional information about the causal influences between these RSNs. Our current findings revealed that a novel network-level mechanism whereby the weaker top-down control flow from DAN results in decreased suppression of VAN and decreased causal flow from AfN to VN, thus further inducing stronger VN activity. Previous studies on brain regions related to loneliness and the causal interaction of the brain networks obtained in the current study suggest that the decreased top-down control from higher networks induced stronger activation in lower sensory networks. Such findings may provide causal interaction evidence in a novel perspective to explain loneliness.

## Supporting information

S1 File (DOCX)
