# Supplementary material for: Causal interactions in resting-state networks predict perceived loneliness
Source: PLoS One. 2017 May 18;12(5):e0177443. doi: 10.1371/journal.pone.0177443 (PMC5436685; doi:10.1371/journal.pone.0177443)
Supplement: S1 File — (DOCX) [file pone.0177443.s001.docx]

**Supporting information**

***Results of one-sample*** ***t-test and two-sample t-test of two groups***

To check the spatial distribution of high-lonely group and low-lonely group, we carried out the one-sample t-test on the four chosen RSNs (i.e. DAN, VAN, AfN and VN) for each group. We also conducted the one-sample t-test on the four chosen RSNs across all the subjects The one-sample t-test results (i.e. Anatomical Locations, hemisphere.) of each group and across all subjects were shown in Tables A-C (all thresholds were set at p<0.05, FDR correction). We found that there were small differences in the spatial distributions among the high-lonely group, low-lonely group and across all the participants. Subsequently, two-sample t-test was applied to find the differences between two groups for each RSN. Table D showed the differences between the spatial distribution of two groups (p<0.05, FDR correction).

***Pearson relationships between loneliness scale and others***

In order to examine whether the relationships among UCLA loneliness scale, State-Trait Anxiety Inventory (STAI), Self-rating depression scale (SDS), Interpersonal Reactivity Index (IRI-C), Trust Scale and Social Support Rating Scale (SSRS) contributed to loneliness, we performed the Pearson correlation between the loneliness scale and the other scales across all participants. Our results suggested that none of these scales were significantly correlated with loneliness scale regardless the high-lonely group or low-lonely group (see Figs A and B).

***Group ICA analysis and four RSNs in two groups***

In order to assess the spatial distributions of four RSNs in high-lonely group and low-lonely group as well as in all subjects, we used the group ICA to select 40 independent components in two groups and all subjects according to the minimum description length criterion. Then, the four RSNs were extracted by a linear template matching process that based on the average z-score of voxels within the RSN template minus the average z-score of voxels outside the template. The components which have the greatest difference were regarded as the interested components (see Figs C-E).

***Loneliness scores and the value of granger causality (AfN🡪VN and DAN🡪VAN)***

To investigate whether the relationships between loneliness scores and GC values were specific to loneliness scales, we examined relationships between several other types of scales (STAI, SDS, IRI-C, Trust Scale and SSRS) and GA values of AfN 🡪VN and DAN 🡪VAN via CGCA analysis. Our results showed that other five scales did not significantly correlate with the GA values in lonely group (Figs F and G). Similarly, the six scales (Loneliness, STAI, SDS, IRI-C, Trust Scale and SSRS) did not correlate with loneliness GA values in non-lonely group (Figs H-I).

**Table A. Brain regions of four RSNs in high-lonely group**

| **Anatomical Locations** | **Hem** | **BA** | **Cluster** | **MNI** | **T value** |
| --- | --- | --- | --- | --- | --- |
|  |  |  | **voxels** | **(x,y,z)** |  |
| ***Dorsal Attention Network (DAN)*** | | | | | |
| Inferior frontal gyrus, triangular | L | 44,45,48 | 114 | -45,14,24 | 13.53 |
|  | R | 44 | 108 | -42,27,15 | 10.96 |
| Middle frontal gyrus | L | 46 | 118 | -48,15,36 | 10.39 |
|  | R | 46 | 102 | 36,36,36 | 14.8 |
| Superior frontal gyrus | L | 8,9,46 | 56 | -24,-9,57 | 15.61 |
|  | R | 9,46 | 42 | 24,30,57 | 15.03 |
| Precentral gyrus | L | 4,6,9 | 68 | -28,-3,60 | 12.05 |
|  | R | 6 | 30 | 51,11,34 | 12.05 |
| Inferior parietal gyrus | L | 2,3,40 | 266 | -31,-54,58 | 12.67 |
|  | R | 2,7,40 | 109 | 29,-57,55 | 16.08 |
| Inferior frontal gyrus, orbital | L | 11,45,47 | 53 | -48,33,9 | 8.59 |
|  | R | 44,48 | 55 | 51,6,24 | 15.27 |
| Postcentral gyrus | L | 43,48 | 100 | -42,-32,48 | 14.83 |
|  | R | 3,43 | 66 | 54,-24,45 | 18.73 |
| Angular | R | 7,40 | 49 | 36,-54,51 | 12.61 |
| Superior parietal gyrus | L | 7,40 | 133 | -30,-54,60 | 12.92 |
|  | R | 7,40 | 81 | 29,-64,50 | 13.42 |
| Supramarginal gryus | L | 2,48,40 | 21 | -55,-38,26 | 7.99 |
|  | R | 2,48 | 30 | 44,-31,42 | 15.81 |
| ***Ventral Attention Network(VAN)*** | | | | | |
| Supra Marginal | R | 2,40,48 | 66 | 54,-46,42 | 11.10 |
| Inferior Parietal | R | 40 | 190 | 54,-57,47 | 17.19 |
| Angular | R | 40 | 261 | 42,-66,39 | 28.24 |
| Superior temporal Gyrus | R | 48 | 20 | 51,-54,21 | 9.91 |
| Middle Temporal Gyrus | R | 21 | 37 | 51,-62,21 | 10.26 |
| Inferior Temporal Gyrus | R | 20 | 41 | 56,-44,-22 | 7.19 |
| Inferior Frontal Gyrus, orbital | L | 47 | 47 | -42,45,-3 | 9.60 |
|  | R | 38,47 | 40 | 42,42,-12 | 8.02 |
| Inferior Frontal Gyrus, triangular | L | 45,48 | 45 | -45,33,15 | 8.93 |
| Inferior Frontal Gyrus, opercular | L | 48 | 11 | -57,5,15 | 9.45 |
| ***Visual Network (VN)*** | | | | | |
| Calcarine fissure | R | 17 | 3 | 24,-93,0 | 14.57 |
| Lingual gyrus | L | 17,18,19 | 35 | -18,-87,-9 | 11.24 |
|  | R | 18,19 | 35 | 18,-87,-9 | 13.73 |
| Fusiform gyrus | L | 19 | 9 | -24,-84,-18 | 9.39 |
|  | R | 18,19 | 37 | 27,-81,-15 | 16.87 |
| Middle occipital gyrus | L | 17,18,19 | 126 | -24,-94,3 | 14.15 |
|  | R | 17,18 | 104 | 27,-95,3 | 12.96 |
| Cuneus | R | 18 | 28 | 24,-96,-9 | 14.86 |
| Superior occipital gyrus | L | 17,18 | 19 | -18,-98,16 | 8.38 |
| ***Affective Network(AfN)*** | | | | | |
| Putamen | L | 48 | 241 | -24,12,-3 | 22.1013 |
|  | R | 48 | 184 | 24,12,0 | 23.18 |
| Pallidum | L | 48 | 74 | -21,-4,0 | 16.31 |
|  | R | 48 | 71 | 21,1,0 | 15.49 |
| Hippocampus | L | 37 | 40 | -30,-13,-15 | 18.15 |
|  | R | 37 | 18 | 27,-9,-15 | 14.26 |
| Amygdala | L | 34,36 | 40 | -24,-3,15 | 14.66 |
|  | R | 28,34 | 46 | 27,0,-15 | 17.99 |
| Caudate | L | 25 | 29 | -12,9.3 | 13.65 |
|  | R | 25 | 27 | 15,9,5 | 12.01 |

Note: Abbreviation: Hem, hemisphere; BA, Brodmann’s area; all p <0.05 with FDR correction

**Table B. Brain regions of four RSNs in low-lonely group**

| **Anatomical Locations** | **Hem** | **BA** | **Cluster** | **MNI** | | **T value** |
| --- | --- | --- | --- | --- | --- | --- |
|  |  |  | **voxels** | **(x,y,z)** | |  |
| ***Dorsal Attention Network (DAN)*** | | | | | | |
| Inferior frontal gyrus, triangular | L | 44,45,48 | 124 | 54,9,30 | | 12.79 |
|  | R | 48 | 78 | 51,30,15 | | 11.15 |
| Middle frontal gyrus | L | 44 | 35 | -24,3,45 | | 9.10 |
|  | R | 44 | 128 | 30,9,60 | | 11.35 |
| Superior frontal gyrus | L | 8,9,46 | 31 | -31,32,40 | | 12.50 |
|  | R | 9,46 | 58 | 18,30,45 | | 12.40 |
| Precentral gyrus | L | 4,6,9 | 101 | -27,-3,57 | | 8.34 |
|  | R | 6 | 21 | 48,7,30 | | 9.73 |
| Inferior parietal gyrus | L | 2,3,40 | 279 | -20,-62,54 | | 17.83 |
|  | R | 2,7,40 | 109 | 42,-39,54 | | 18.11 |
| Inferior frontal gyrus, orbital | L | 11,45,47 | 44 | -39,6,21 | | 7.25 |
|  | R | 44,48 | 60 | 54,9,30 | | 17.80 |
| Superior occipital gyrus | R | 7,19 | 11 | -25,-66,38 | | 8.78 |
| Postcentral gyrus | L | 40 | 67 | -42,-37,57 | | 13.20 |
|  | R | 3,43 | 44 | 43,-29,44 | | 13.35 |
| Angular | R | 7,40 | 49 | -33,-65,43 | | 10.06 |
| Superior parietal gyrus | L | 7,40 | 115 | -24,-54,60 | | 12.26 |
|  | R | 7,40 | 81 | 27,-69,51 | | 13.10 |
| Supramarginal gryus | L | 2,48,40 | 22 | -51,-24,41 | | 7.03 |
|  | R | 2,48 | 30 | 42,-32,42 | | 13.10 |
| ***Ventral Attention Network(VAN)*** | | | | | | |
| Supra Marginal | R | 2,40,48 | 58 | | 57,-46,41 | 11.62 |
| Inferior Parietal | R | 40 | 104 | | 58,-36,45 | 11.72 |
| Angular | R | 40 | 235 | | 42,-60,39 | 29.71 |
| Superior temporal gyrus | R | 48 | 18 | | 57,-58,21 | 11.2 |
| Middle Temporal Gyrus | R | 21 | 48 | | 51,-62,21 | 12.70 |
| Inferior Temporal Gyrus | R | 20 | 33 | | 57,-39,-21 | 7.79 |
| Inferior Frontal Gyrus, orbital | L | 47 | 33 | | -33,33,-12 | 9.58 |
|  | R | 38,47 | 27 | | 51,18,-12 | 8.31 |
| Inferior Frontal Gyrus,triangular | L | 45,48 | 32 | | -45,27,21 | 8.63 |
| Inferior Frontal Gyrus, opercular | L | 48 | 16 | | -42,15,18 | 7.60 |
| ***Visual Network (VN)*** | | | | | | |
| Calcarine fissure | R | 17 | 3 | | 25,-96,3 | 14.40 |
| Lingual gyrus | L | 17,18,19 | 21 | | -15,-87,-9 | 15.94 |
|  | R | 18,19 | 35 | | 21,-90,-12 | 13.99 |
| Fusiform gyrus | L | 19 | 9 | | -24,-78,-12 | 7.18 |
|  | R | 18,19 | 37 | | 27,-87,-12 | 14.65 |
| Middle occipital gyrus | L | 17,18,19 | 122 | | -18,-96,9 | 14.52 |
|  | R | 17,18 | 93 | | 27,-96,9 | 14.70 |
| Cuneus | R | 18 | 29 | | 24,-96,12 | 15.68 |
| Superior occipital gyrus | L | 17,18 | 10 | | -18,-99,15 | 9.83 |
| ***Affective Network(AfN)*** | | | | | | |
| Putamen | L | 48 | 208 | | -15,12,0 | 20.30 |
|  | R | 48 | 132 | | 21,9,-3 | 19.54 |
| Pallidum | L | 48 | 66 | | -18,3,0 | 13.88 |
|  | R | 48 | 55 | | 21,4,0 | 18.03 |
| Amygdala | L | 34,36 | 49 | | -24,-2,-18 | 12.33 |
|  | R | 28,34 | 36 | | 27,0,-18 | 12.40 |
| Hippocampus | L | 34 | 20 | | -27,-10,-18 | 12.01 |
|  | R | 34 | 27 | | 27,-11,14 | 14.38 |
| Caudate | L | 25 | 40 | | -9,12,-3 | 13.80 |
|  | R | 25 | 63 | | 12,15,-1 | 15.32 |

Note: Abbreviation: Hem, hemisphere; BA, Brodmann’s area; all p <0.05 with FDR correction

**Table C. Brain regions of four RSNs across all subjects**

| **Anatomical Locations** | **Hem** | **BA** | **Cluster** | **MNI** | **T value** |
| --- | --- | --- | --- | --- | --- |
|  |  |  | **voxels** | **(x,y,z)** |  |
| ***Dorsal Attention Network (DAN)*** | | | | | |
| Inferior frontal gyrus, triangular | L | 44,45,48 | 186 | -45,30,21 | 16.38 |
|  | R | 44 | 18 | 51,14,23 | 13.36 |
| Middle frontal gyrus | L | 46 | 48 | -48,15,36 | 10.39 |
|  | R | 46 | 23 | 45,33,21 | 13.98 |
| Superior frontal gyrus | L | 8,9,46 | 68 | -31,32,40 | 12.22 |
|  | R | 9,46 | 63 | 34,31,38 | 10.19 |
| Precentral gyrus | L | 4,6,9 | 121 | -45,6,33 | 11.65 |
|  | R | 6 | 23 | 48,7,30 | 10.88 |
| Inferior parietal gyrus | L | 2,3,40 | 288 | -20,-62,54 | 22.83 |
|  | R | 2,7,40 | 115 | 40,-43,51 | 20.72 |
| Inferior frontal gyrus, orbital | L | 11,45,47 | 68 | -39,36,-3 | 9.58 |
|  | R | 44,48 | 65 | 51,9,30 | 18.32 |
| Postcentral gyrus | L | 43,48 | 79 | -51,-33,51 | 14.86 |
|  | R | 3,43 | 44 | 47,-27,42 | 13.28 |
| Angular | R | 7,40 | 49 | -33,-65,43 | 13.74 |
| Superior parietal gyrus | L | 7,40 | 92 | -27,-57,45 | 10.26 |
|  | R | 7,40 | 87 | 33,62,2 | 18.88 |
| Supramarginal gryus | L | 2,48,40 | 41 | -53,-26,42 | 9.68 |
|  | R | 2,48 | 30 | 56,-23,42 | 11.18 |
| ***Ventral Attention Network(VAN)*** | | | | | |
| Supra Marginal | R | 2,40,48 | 237 | 63,-30,30 | 16.66 |
| Inferior Parietal | R | 40 | 144 | 58,-36,45 | 11.72 |
| Angular | R | 40 | 15 | 57,-51,33 | 18.65 |
| Superior temporal Gyrus | R | 48 | 11 | 63,-24,15 | 13.28 |
| Middle Temporal Gyrus | R | 21 | 34 | 69,-30,-12 | 16.25 |
| Inferior Temporal Gyrus | R | 20 | 30 | 56,-44,-22 | 15.46 |
| Inferior Frontal Gyrus, orbital | L | 47 | 100 | -51,27,-6 | 13.33 |
|  | R | 38,47 | 21 | 51,27,-9 | 9.28 |
| Inferior Frontal Gyrus, triangular | L | 45,48 | 49 | -46,25,1 | 11.88 |
| Inferior Frontal Gyrus, opercular | L | 48 | 237 | 63,-30,30 | 16.66 |
| ***Visual Network (VN)*** | | | | | |
| Calcarine fissure | R | 17 | 3 | 25,-101,1 | 6.89 |
| Lingual gyrus | L | 17,18,19 | 35 | -9,-75,3 | 13.16 |
|  | R | 18,19 | 59 | 18,-85,-12 | 11.18 |
| Fusiform gyrus | L | 19 | 11 | -32,-71,-16 | 10.1 |
|  | R | 18,19 | 37 | 23,-81,-13 | 16.28 |
| Middle occipital gyrus | L | 17,18,19 | 141 | -27,-93,9 | 15.22 |
|  | R | 17,18 | 153 | 24,-93,3 | 8.83 |
| Cuneus | R | 18 | 47 | 15,-96,21 | 10 |
| Superior occipital gyrus | L | 17,18 | 30 | -20,-98,16 | 13.53 |
| ***Affective Network(AfN)*** | | | | | |
| Putamen | L | 48 | 239 | -22,4,1 | 18.23 |
|  | R | 48 | 69 | 21,6,-3 | 16.66 |
| Pallidum | L | 48 | 72 | -18,6,1 | 14.58 |
|  | R | 48 | 65 | 21,6,-3 | 13.69 |
| Hippocampus | L | 37 | 49 | -28,-6,-16 | 19.86 |
|  | R | 37 | 60 | 22,1,-20 | 21.35 |
| Amygdala | L | 34,36 | 239 | -22,4,1 | 18.23 |
|  | R | 28,34 | 69 | 21,6,-3 | 16.66 |
| Caudate | L | 25 | 29 | -12,9.3 | 13.65 |
|  | R | 25 | 10 | 15,9,5 | 12.01 |
| Caudate | L | 25 | 29 | -12,9.3 | 13.65 |
|  | R | 25 | 10 | 15,9,5 | 12.01 |

Note: Abbreviation: Hem, hemisphere; BA, Brodmann’s area; all p <0.05 with FDR correction

**Table D. Different Brain regions of four RSNs between high-lonely group and low-lonely group measured by two-sample t test**

| **Anatomical Locations** | **Hem** | **BA** | **Cluster** | **MNI** | **T value** |
| --- | --- | --- | --- | --- | --- |
|  |  |  | **voxels** | **(x,y,z)** |  |
| ***Dorsal Attention Network (DAN)*** | | | | | |
| Precentral gyrus | L | 4,6,9 | 28 | -42,0,30 | 2.36 |
| Superior parietal gyrus | L | 7,40 | 112 | -24,-66,51 | 3.60 |
|  | R | 2,48 | 30 | 30,-63,51 | 2.53 |
| ***Affective Network(AfN)*** | | | | | |
| Pallidum | L | 48 | 10 | -21,-9,3 | 1.76 |
| Hippocampus | L | 25 | 49 | -27,-27,-12 | 3.42 |
| ***Ventral Attention Network(VAN)*** | | | | | |
| Supra Marginal | R | 2,40,48 | 46 | 51,-39,31 | 2.11 |
| Inferior Parietal | R | 40 | 53 | 51,-48,45 | 2.90 |
| Angular | R | 40 | 11 | 33,-60,36 | 2.70 |
| Inferior Frontal Gyrus, triangular | L | 45,48 | 22 | -45,33,12 | 2.89 |
| ***Visual Network (VN)*** | | | | | |
| Inferior Occipital Gyrus | R | 18,19 | 47 | 33,-87,-9 | 2.66 |
| Middle occipital gyrus | R | 17,18,19 | 35 | 36,-87,0 | 1.90 |

Note: All p <0.05 with FDR-correction. If p<0.001, FDR-correction applied. There was no difference in the RSNs between high-lonely group and low-lonely group.


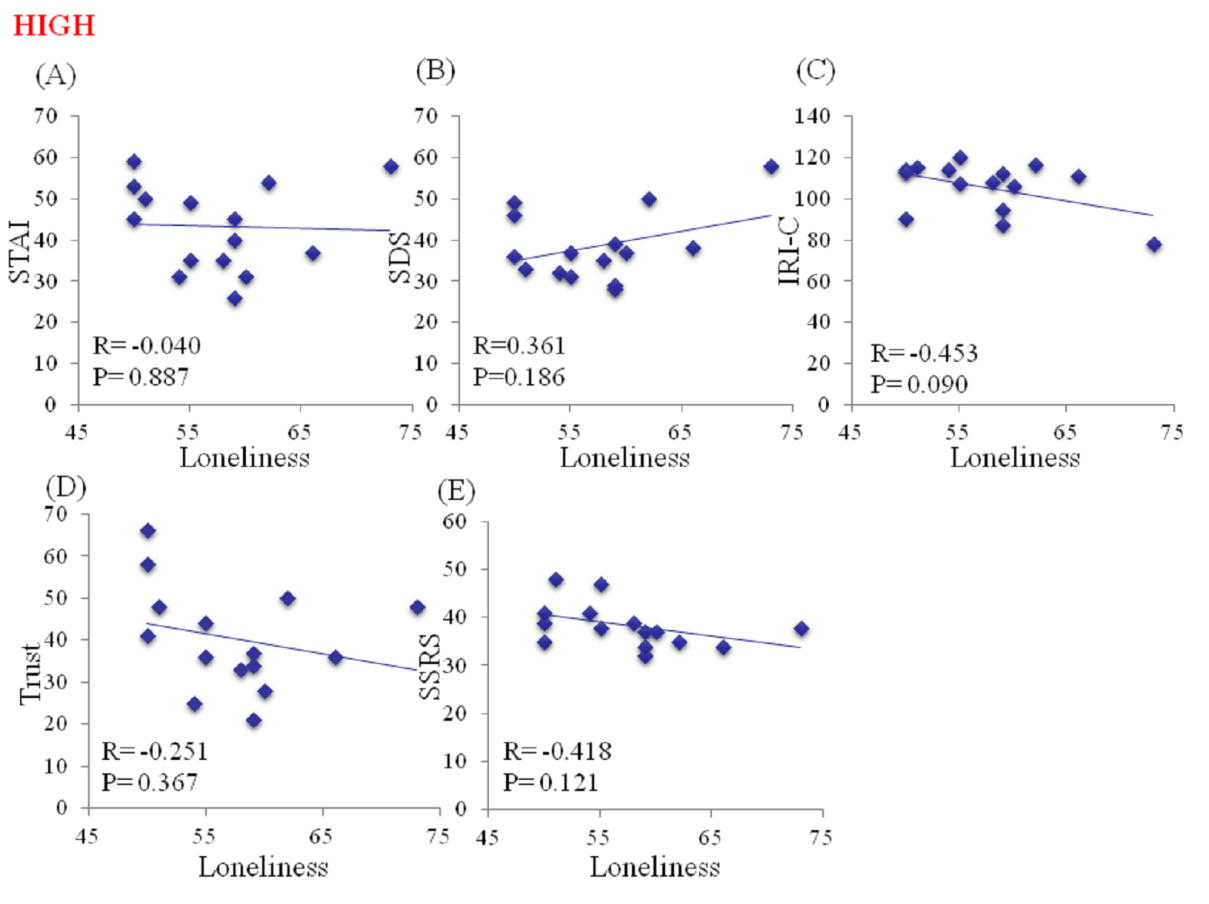


**Figure A**. Relationships between Loneliness Scale and other five scales (n = 15) in high-lonely group. (A) State-Trait Anxiety Inventory (STAI), (B) Self-rating depression scale (SDS), (C) Interpersonal Reactivity Index (IRI-C), (D) Trust Scale and (E) Social Support Rating Scale (SSRS).


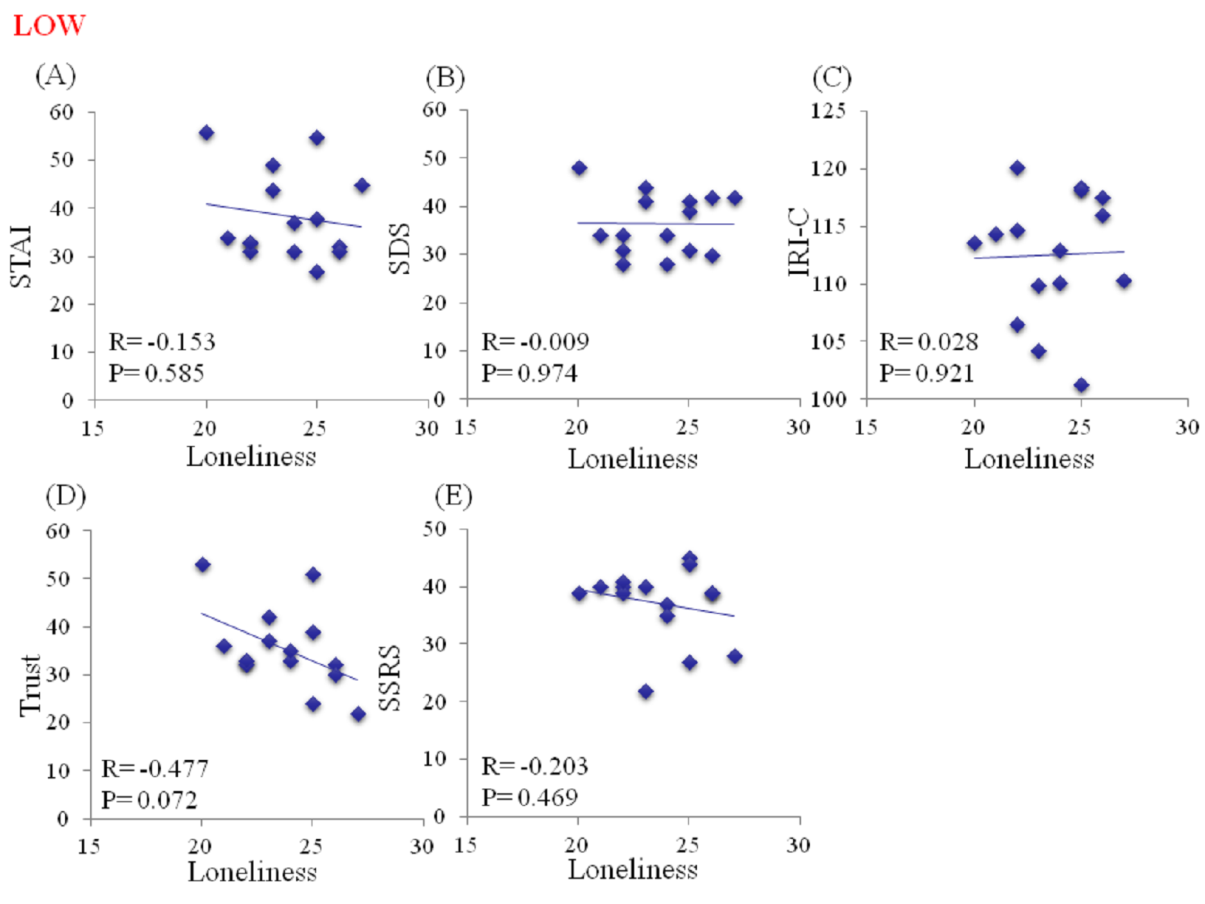


**Figure B.** Relationships between Loneliness Scale and other five scales (n = 15) in low-lonely group. (A) State-Trait Anxiety Inventory (STAI), (B) Self-rating depression scale (SDS), (C) Interpersonal Reactivity Index (IRI-C), (D) Trust Scale and (E) Social Support Rating Scale (SSRS).


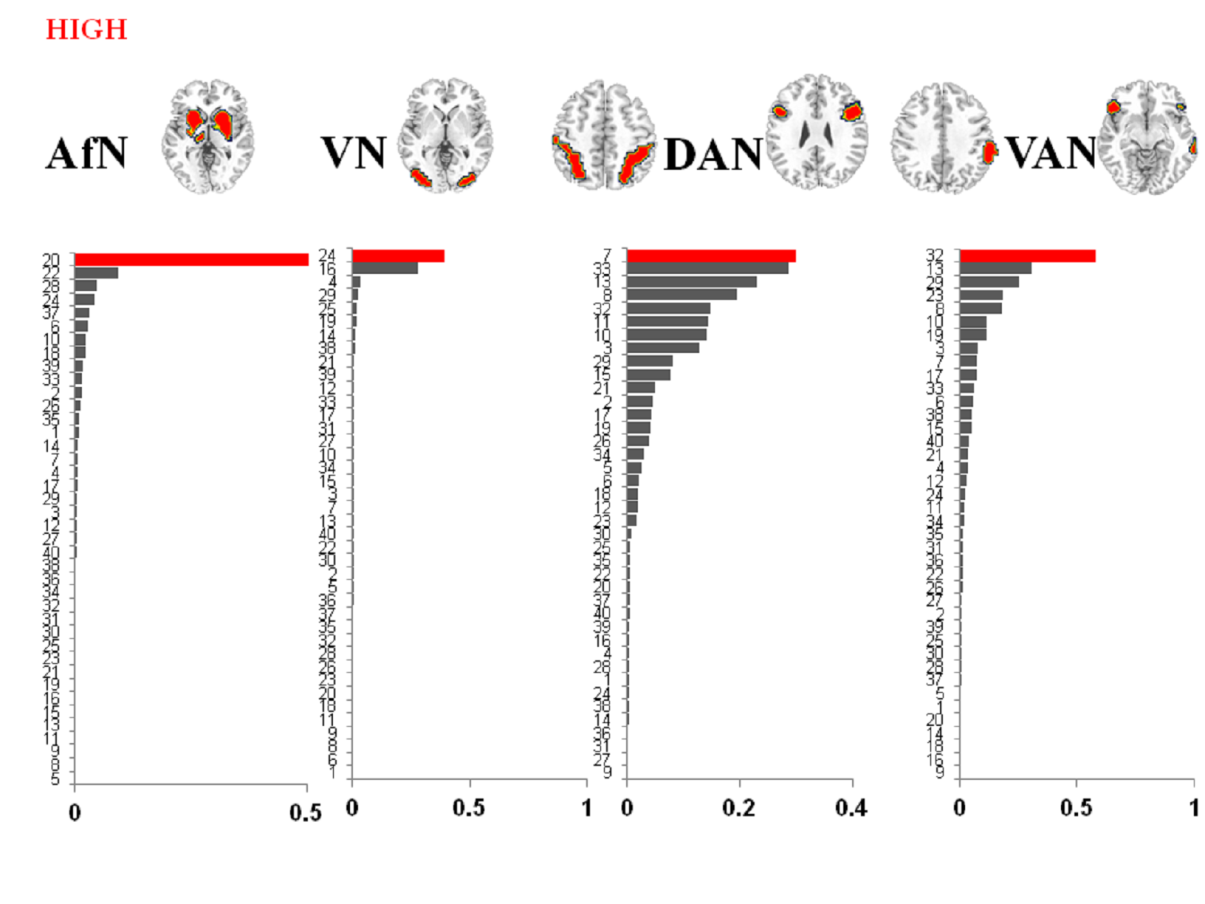


**Figure C.** The spatial correlation coefficients of the 40 ICs of high-lonely group with respect to the four resting state network (RSN) templates. The templates for affective network (AfN), visual network (VN), dorsal attention network (DAN), and ventral attention network (VAN) were obtained from functional imaging in neuropsychiatric disorders lab (http://findlab.stanford.edu/functional_ROIs.html). Our selected RSNs corresponded to the ICs (it is colored in red) with the largest correlations with the templates.


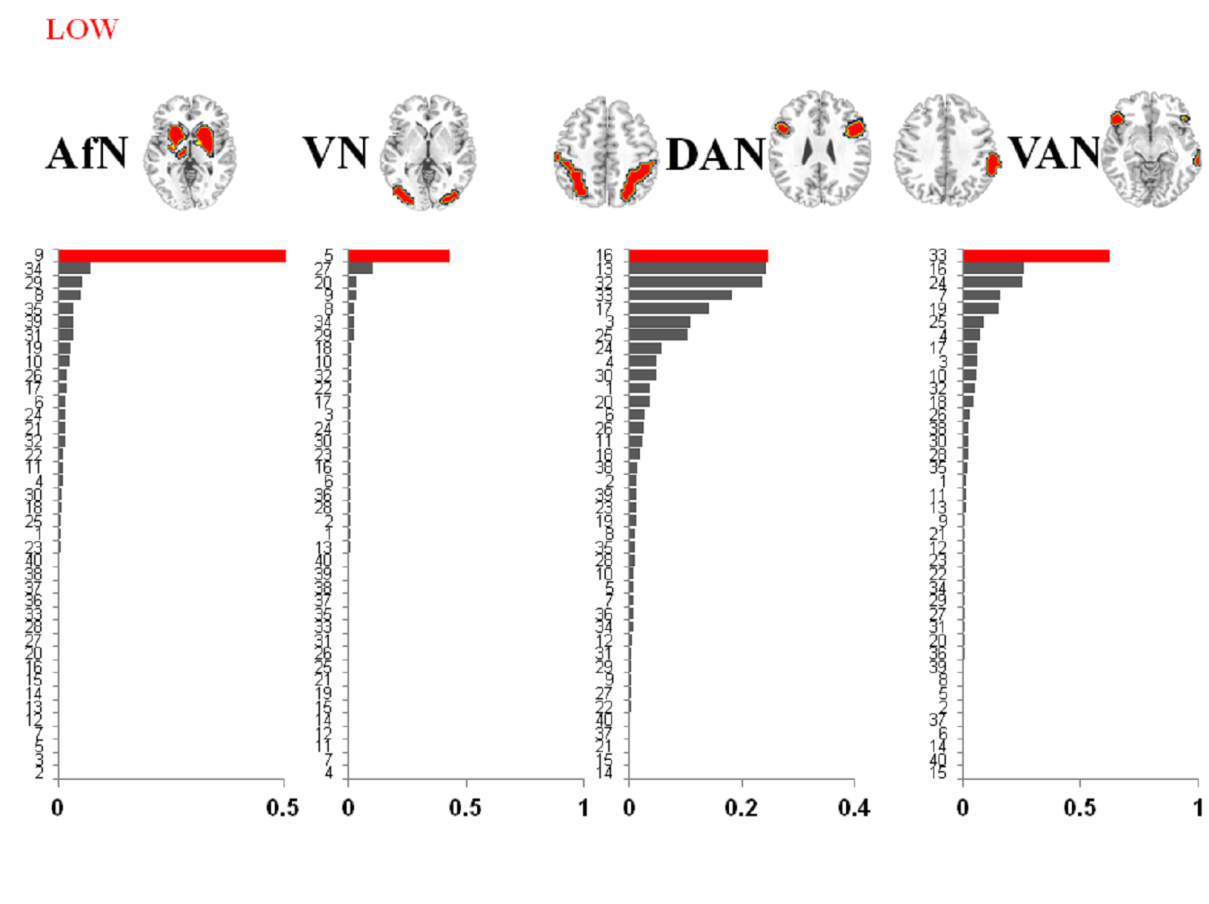


**Figure D.** The spatial correlation coefficients of the 40 ICs of low-lonely group with respect to the four resting state network (RSN) templates. The templates for affective network (AfN), visual network (VN), dorsal attention network (DAN), and ventral attention network (VAN) were obtained from functional imaging in neuropsychiatric disorders lab (http://findlab.stanford.edu/functional_ROIs.html). Our selected RSNs corresponded to the ICs (it is colored in red) with the largest correlations with the templates.


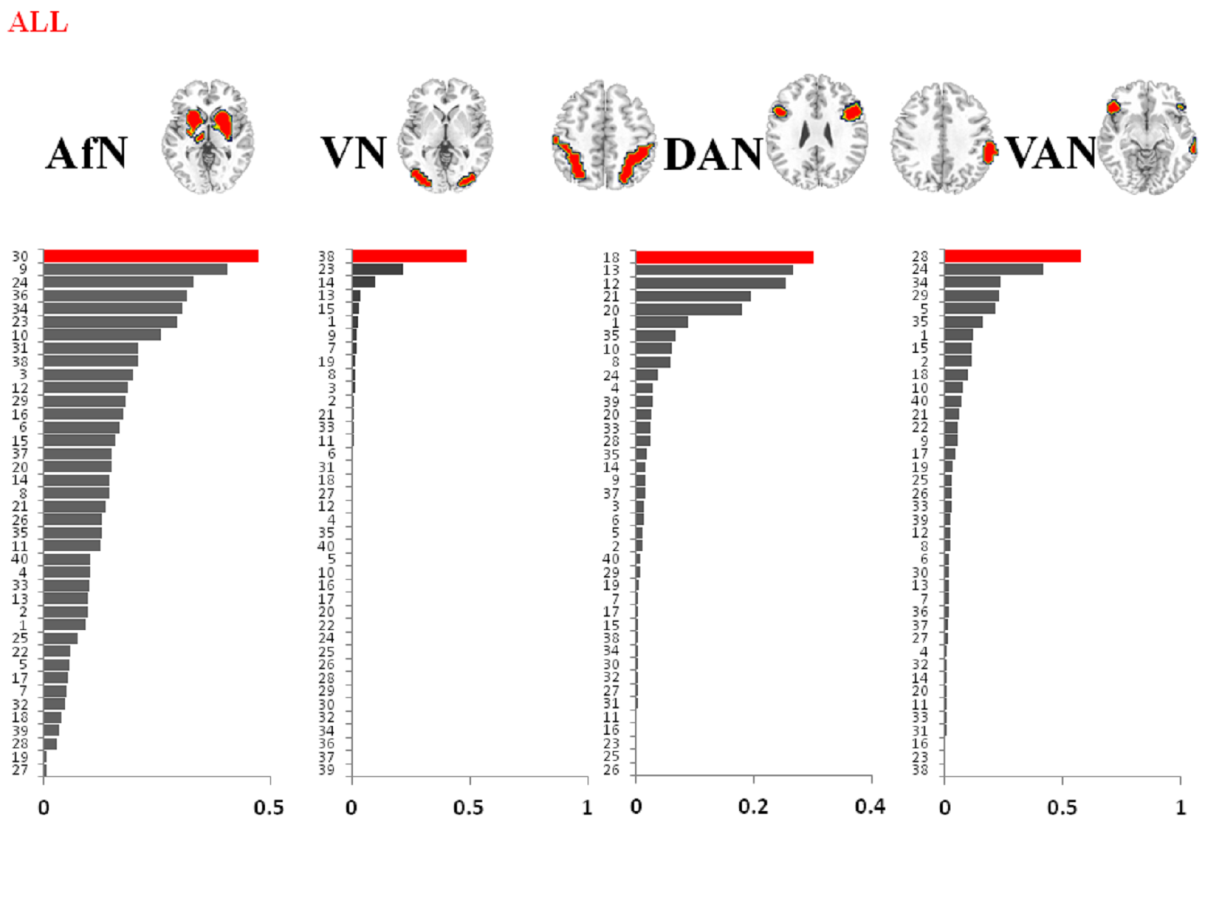


**Figure E.** The spatial correlation coefficients of the 40 ICs of all subjects with respect to the four resting state network (RSN) templates. The templates for affective network (AfN), visual network (VN), dorsal attention network (DAN), and ventral attention network (VAN) were obtained from functional imaging in neuropsychiatric disorders lab (<http://findlab.stanford.edu/functional_ROIs.html>). Our selected RSNs corresponded to the ICs (it is colored in red) with the largest correlations with the templates.


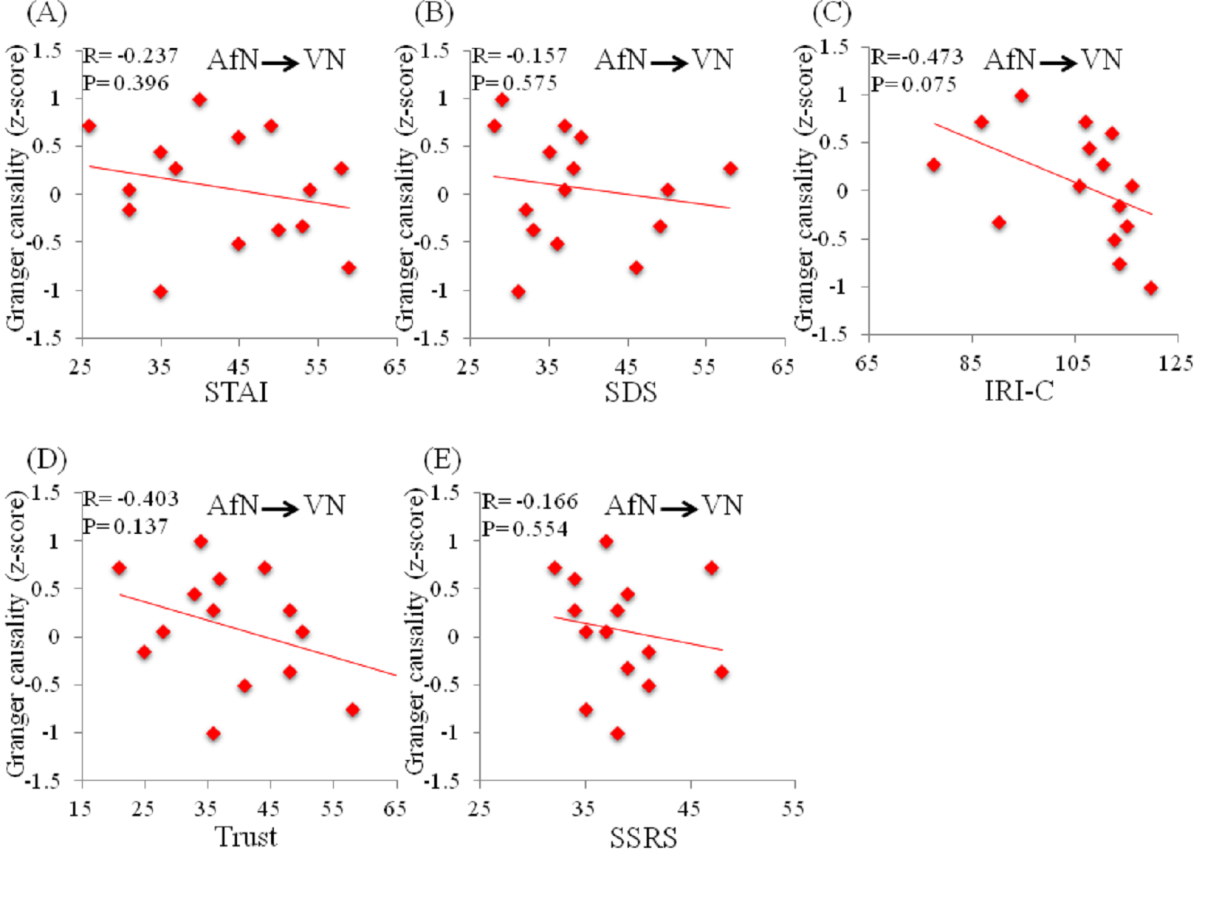


**Figure F.** Correlation relationships between different scales and GC value (AfN🡪VN) in lonely group. (A) State-Trait Anxiety Inventory (STAI), (B) Self-rating depression scale (SDS), (C) Interpersonal Reactivity Index (IRI-C), (D) Trust Scale and (E) Social Support Rating Scale (SSRS). All Pearson’s correlations did not reach statistical significance (p<0.05, FDR correction).


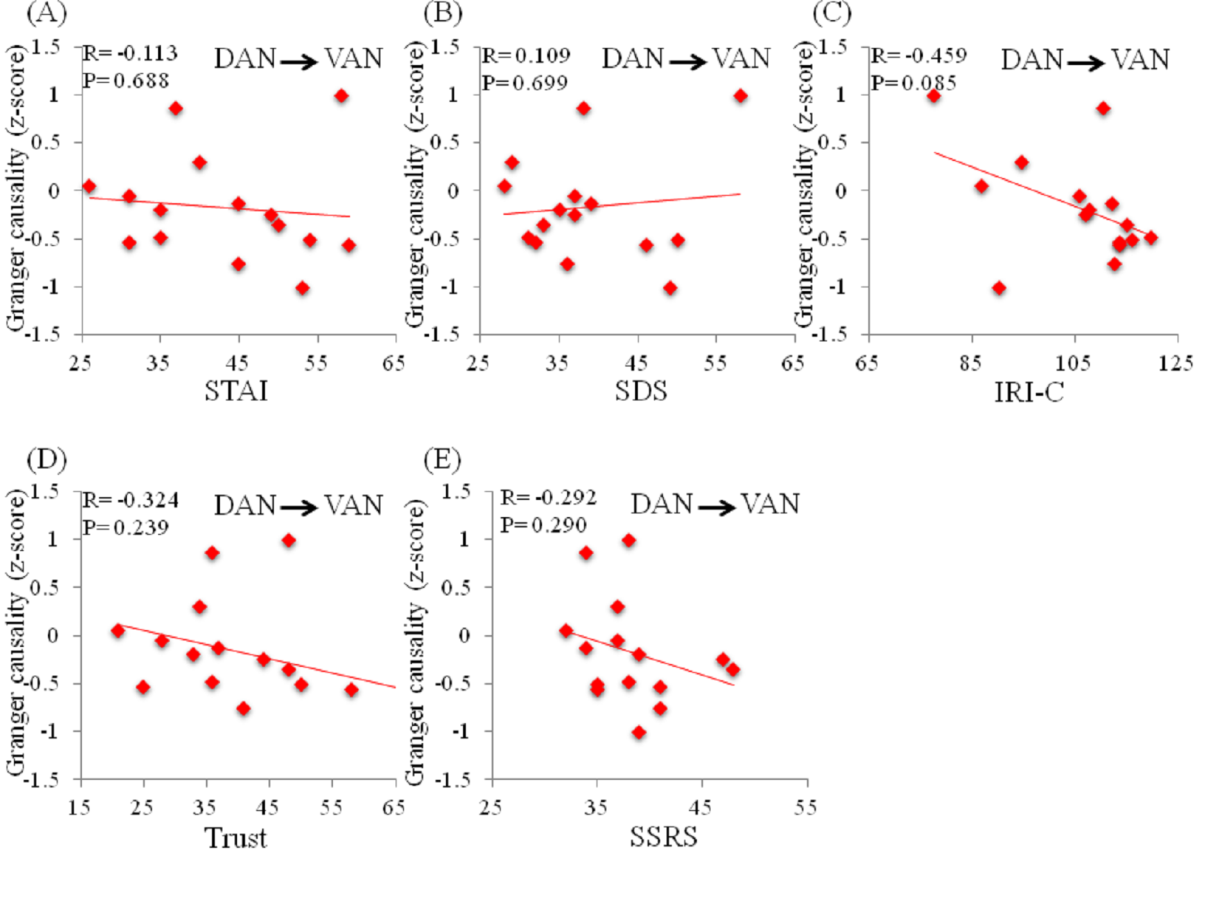


**Figure G.** Correlation relationships between different scales and GC value (DAN🡪VAN) in lonely group. (A) State-Trait Anxiety Inventory (STAI), (B) Self-rating depression scale (SDS), (C) Interpersonal Reactivity Index (IRI-C), (D) Trust Scale and (E) Social Support Rating Scale (SSRS). All Pearson’s correlations did not reach statistical significance (p<0.05, FDR correction).


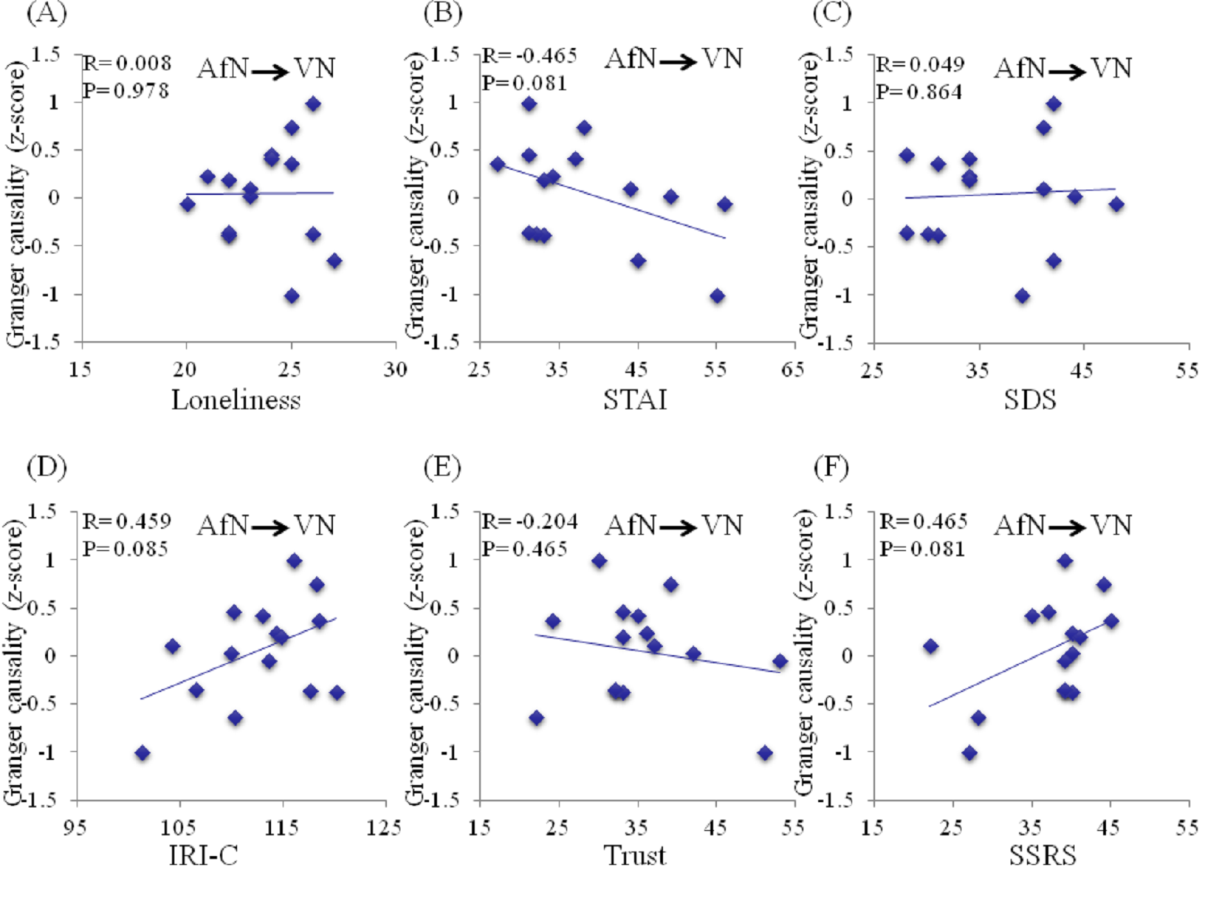


**Figure H.** Correlation relationships between different scales and GC value (AfN🡪VN) in non-lonely group. (A) UCLA loneliness scale (B) State-Trait Anxiety Inventory (STAI), (C) Self-rating depression scale (SDS), (D) Interpersonal Reactivity Index (IRI-C), (E) Trust Scale and (F) Social Support Rating Scale. All Pearson’s correlations did not reach statistical significance (p<0.05, FDR correction).


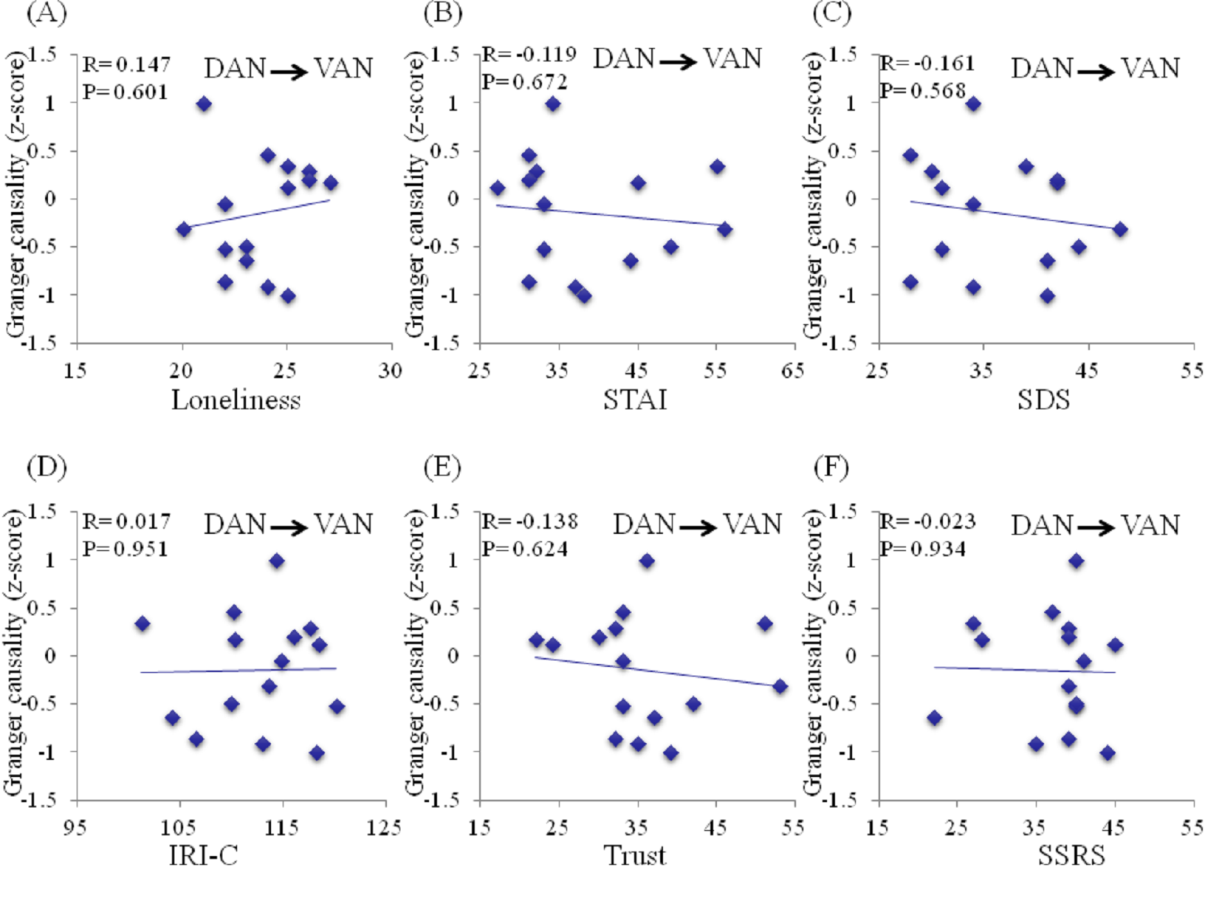


**Figure I.** Correlation relationships between different scales and GC value (DAN🡪VAN) in non-lonely group. (A) UCLA loneliness scale (B) State-Trait Anxiety Inventory (STAI), (C) Self-rating depression scale (SDS), (D) Interpersonal Reactivity Index (IRI-C), (E) Trust Scale and (F) Social Support Rating Scale. All Pearson’s correlations did not reach statistical significance (p<0.05, FDR correction).
